# The values of elastic quantitative and semi-quantitative indexes measured from different frequencies in the establishment of prediction models for breast tumor diagnosis

**DOI:** 10.1186/s12880-022-00915-1

**Published:** 2022-11-15

**Authors:** Xiao Xie, Yibo Ma, Xiaoxiao Xing, Haixia Zhou, Shuiqing Liu, Yanyan Zhang, Min Xu

**Affiliations:** 1 Department of Ultrasound Medicine, the First People’s Hospital of Changzhou and The Third Affiliated Hospital of Soochow University, 213003 Changzhou, China; 2 Department of Cardiology, the First People’s Hospital of Changzhou and The Third Affiliated Hospital of Soochow University, 213003 Changzhou, China

**Keywords:** Breast, Ultrasound, Elastography, Prediction model, Frequency

## Abstract

**Objective:**

To obtain the elastic quantitative and semi-quantitative indexes of solid breast masses using ultrasound linear array probes with two different frequencies, and to construct prediction models and evaluate their diagnostic values.

**Methods:**

A total of 92 patients who were scheduled for surgical treatment on solid breast masses were enrolled in this study. Linear array probes with two frequencies, 9-3 MHz (L9 group) and 14-5 MHz (L14 group), were used for sound touch elastography and strain elastography before surgery, and the maximum elasticity value (Emax), average elasticity value (Emean), minimum elasticity value (Emin), standard deviation (SD)(in kPa), elasticity ratio (E), and strain ratio to fat (SRf) were recorded and calculated for the breast mass (A) and surrounding tissues (Shell). The elastic characteristic indexes of the L9 group and L14 group were compared, and the prediction models of these two groups were constructed using Logistic regression method.

**Results:**

The diagnostic performance of the prediction model based on L9 group was better than the model based on L14 group (AUC: 0.904 vs. 0.810, P = 0.0343, z = 2.116) and the best single index EMax-shell-L9 (P = 0.0398, z = 2.056). The sensitivity of L9 based model was 85.19% and the specificity was 84.21%.

**Conclusion:**

The prediction model based on quantitative and semi-quantitative elastic ultrasound indexes from L9-3 probe exhibited better performance, which could improve the diagnostic accuracy for malignant breast tumors.

**Supplementary Information:**

The online version contains supplementary material available at 10.1186/s12880-022-00915-1.

## Introduction

Elastography is widely used in ultrasound examination, and breast tissue is the most studied type of tissue, in which elastography has shown significant value for disease diagnosis [1]. The influence of ultrasound frequency and depth on the measured value of elastic ultrasound has been reported in in vitro experiments, and most of them compared convex array probes and linear array probes [2, 3]. However, there are few reports on the diagnostic value of linear array probes with different frequencies for breast masses in clinical applications. The commonly used frequency of breast ultrasound scan in clinic is 12-14 MHz, which gives good clarity. However, in elastography, the quality of elastic image is sometimes poor, especially the shear wave elastography, which causes great deal of variation between examiners [4, 5]. Examiners are often faced with confusion about how to choose a better probe to complete an elastic ultrasound examination. Therefore, exploring the best frequency of breast elastic ultrasound is highly important for improving its diagnostic value. Previous research results showed that objective and quantitative imaging indexes could effectively reflect the prognosis or prediction of the disease [6–8]. Moreover, the diagnosis based on a combination of multiple indexes is more promising than the diagnosis based on a single index, and it is good enough to affect clinical decisions. However, there is multi-dimensional collinearity among some elasticity indexes; thus, the factors with clinical significance may be screened out during the traditional logistics regression process. LASSO regression is a reduction method of linear regression. It can filter and establish an algorithm based on each elasticity index to achieve variable selection and parameter estimation [9]. There are emerging computation methods based on R language that can automatically extract high-dimensional features from various types of data, perform data mining and analysis, and provide decision support. In this study, we used linear array probes with different frequencies of the same ultrasound instrument to perform shear-wave elastography and strain elastography on the same group of breast masses, which were diagnosed with pathological examination. We obtained multiple quantitative and semi-quantitative indexes, and constructed prediction models at different frequencies using R. The performance of different models was compared to identify the frequency that was more useful for breast elastic ultrasound, and establish the elastic ultrasound prediction model that can better distinguish benign and malignant breast tumors.

## Materials and methods

### Patient selection

From July 2017 to December 2018, 129 female patients with breast lesions identified by clinical examination and/or imaging underwent conventional ultrasound (US) and US elastography consecutively at Changzhou First People’s Hospital. This retrospective study was approved by the Ethics Committee of Changzhou First People’s Hospital [(2018) Section No. 054], and informed consent was obtained from all patients. The inclusion criteria were: (1) The solid masses were classified based on BI-RADS; (2) Two linear array probes with 9 − 3 MHz and 14 − 5 MHz were used for sound touch elastography and strain elastography; (3) For the masses from the same patient and with same pathological diagnosis, the bigger mass was selected; (4) Pathological results are obtained by surgical resection of all lesions. Exclusion criteria: (1) The pathological result was benign simple cyst; (2) Poor elasticity images; (3) The patient received neoadjuvant chemotherapy; (4) nodular cystic fat necrosis (fat necrosis). Totally, we measured 134 breast lesions. Forty-two lesions in 36 patients were excluded later. Finally, 92 breast lesions in 91 consecutive women patients (mean age, 46.14 ± 13.26 years; range, 24–83 years) were included in the final data analysis (Figure S1). In these patients, one person had two lesions, one benign and one malignant.

### Image acquisition and interpretation

The Mindray RESONA 7OB ultrasonic diagnostic instrument (Mindray Medical International, Shenzhen, Guangdong Province, China) was used in this study. We used linear array probes with two frequencies: 9-3 MHz and 14-5 MHz, both of which were able to perform strain elastography and sound touch elastography (STE).

Routine ultrasound examination and BI-RADS classification were performed on breast masses before surgery, and then elastography examination was conducted on the area of interest (tumor area). Elastography examination included strain elastography and sound touch elastography at two frequencies. The same mass slice was used for imaging and the imaging frame was in same size and position. The elastography image was stored as a stable dynamic image of 3 s. Two ultrasound physicians selected three static images from the moving image for analysis without knowing the content of the ultrasound report and the pathology report. The elastic value measurement of all images was done on an online ultrasonic instrument. A represents the elasticity value of the mass. Considering the uniformity when using fat as the reference for normal tissues [10, 11], the B of control tissue used the soft blue part of the fat layer. The elastic ultrasound data included the elastic strain ratio (SR) (A/Shell, B/A, and B/Shell) obtained by the strain elasticity, where fat was used as a reference for B (represented as SRf). Sound touch elastography can yield the elastic values (Mean, Max, Min, SD) (unit kPa) of the mass (A), the 2 mm shell surrounding mass (Shell), and the elasticity ratio (E) between A, Shell and the glands of the same level (B) (Figure S2). The elastic images were analysed without knowing the histopathological results of the lesion. All parameters were analyzed by two attending doctors with more than 8 years of experience, and the average value was taken. The two doctors were blinded to each other, and did not know the results of the ultrasound report. The pathological results of the masses at 1–2 weeks after surgery were recorded.

Elasticity quality control requirements: sound touch elastography used the machine’s built-in reliability monitoring, and the reliability must be greater than 90%. The energy column of the strain elastography must exceed the dark blue part, and the appearance of a dark blue stripe between fat and gland in the image was required.

### Statistical analysis

All statistical tests were performed using R 3.4.1 (http://www.R-project.org/). The “glmnet” package was used for LASSO regression model analysis, the “pROC” package was used to plot ROC curves, and the “rms” package was used for Nomogram construction. Measurement data were expressed as mean ± standard deviation, and count data were expressed as percentage or rate. When applicable, the unpaired Student-t test or Mann-Whitney nonparametric test was used to compare between continuous variables. Comparison between categorical variables were performed using Pearson’s chi-square test and Fisher’s exact test.

In this study, we used the least absolute shrinkage and selection operator (LASSO) regression, which is suitable for the regression of high-dimensional data. Using LASSO algorithm and the 10-fold cross-validation parameter adjustment method, we selected the elasticity indexes with non-zero coefficients from the two groups of elastic quantitative and semi-quantitative indexes, and weighed them by the respective LASSO coefficients to obtain a linear combination formula of the selected indexes. Then, we used the formula to calculate the malignancy risk score of each lesion to reflect the malignancy risk of the mass. After that, the receiver operating characteristic (ROC) curve for each model was plotted, and the diagnostic efficacy of the area under the curve (AUC) of different models was compared using the method from DeLong et al. [12]. All statistical tests were two-sided, and P ≤ 0.05 was considered statistically significant.

## Results

### General information

The average age of the patients was 46.14 ± 13.26 years old, the averaged long diameter of the lesion was 1.85 ± 0.84 cm, and the averaged short diameter was 1.10 ± 0.53 cm. Postoperative pathology showed that, among 92 cases, 39 cases were malignant (42.39%), of which 27 cases were invasive ductal carcinoma (29.35%), 5 cases were intraductal carcinoma (5.43%), 4 cases were invasive ductal carcinoma with partial intraductal carcinoma ( 4.35%), 1 case was mucinous carcinoma (1.09%), 1 case was sarcomatoid carcinoma (1.09%), and 1 case was solid papillary carcinoma (1.09%). There were 53 cases with benign lesions (57.61%), of which 40 cases were fibroadenoma (43.48%), 4 cases were intraductal papilloma (4.35%), 2 case was phyllodes tumor (2.17%), 5 cases were adenopathy (5.43%), and 2 cases were dysplasia (2.17%).

A total of 18 elastic quantitative and semi-quantitative indexes measured with the L14 and L9 probes were collected for each mass before operation, and the pathological results were used as the diagnosis reference. The comparison of the 18 indexes in L14 and L9 groups is shown in Table 1. The range of intra-observer ICCs was 0.842 ~ 0.970, and the range of inter-observer ICCs was 0.835 ~ 0.953, which were both > 0.75, indicating a good repeatability within and between observers.

**Table 1:** Comparison of the elastic indexes of breast mass between L14-5 probe and L9-3 probe before operation

Pathology	Benign (n = 53)	Malignant (n = 39)	*P*-value
Age	40.07 ± 10.05	56.26 ± 11.52	< 0.001
EMean-A-L14	29.14 ± 11.06	35.89 ± 10.30	0.004
EMax-A-L14	80.04 ± 44.94	105.16 ± 44.01	0.009
Emin-A-L14	9.05 ± 5.35	10.54 ± 6.67	0.237
ESD-A-L14	11.26 ± 4.56	14.00 ± 5.36	0.010
EMean-Shell-L14	29.59 ± 11.95	40.27 ± 11.32	< 0.001
EMax-Shell-L14	83.10 ± 34.67	120.02 ± 50.03	< 0.001
EMin-Shell-L14	7.88 ± 5.81	10.53 ± 5.88	0.035
ESD-Shell-L14	13.74 ± 5.43	17.30 ± 6.29	0.005
EMean-Shell/A-L14	1.72 ± 4.20	2.49 ± 6.19	0.480
EMax-Shell/A-L14	1.55 ± 2.81	1.42 ± 0.97	0.795
EMin-Shell/A-L14	0.95 ± 0.60	1.12 ± 0.79	0.225
ESD-Shell/A-L14	1.35 ± 0.49	1.30 ± 0.33	0.583
E-Shell/A-L14	1.04 ± 0.19	1.15 ± 0.19	0.006
E-A/B-L14	2.31 ± 1.02	2.00 ± 0.91	0.150
E-Shell/B-L14	2.33 ± 1.04	2.28 ± 1.02	0.814
SRf-A/Shell-L14	0.72 ± 0.19	0.66 ± 0.24	0.169
SRf-B/A-L14	3.25 ± 1.09	4.16 ± 1.83	0.004
SRf-B/Shell-L14	2.29 ± 0.75	2.57 ± 1.11	0.113
SRf-A/Shell-L9	0.71 ± 0.26	0.66 ± 0.22	0.356
SRf-B/A-L9	2.84 ± 1.01	4.10 ± 1.24	< 0.001
SRf-B/Shell-L9	2.03 ± 0.74	2.67 ± 0.89	< 0.001
EMean-A-L9	23.63 ± 12.79	31.23 ± 14.91	0.010
EMax-A-L9	87.02 ± 52.51	161.86 ± 91.40	< 0.001
EMin-A-L9	5.23 ± 2.86	4.46 ± 2.56	0.189
ESD-A-L9	12.58 ± 7.36	20.73 ± 12.59	< 0.001
EMean-Shell-L9	23.80 ± 14.28	40.33 ± 17.25	< 0.001
EMax-Shell-L9	92.19 ± 62.44	190.73 ± 91.62	< 0.001
EMin-Shell-L9	4.27 ± 2.38	4.48 ± 2.69	0.687
ESD-Shell-L9	15.16 ± 11.46	29.20 ± 17.37	< 0.001
EMean-Shell/A-L9	0.98 ± 0.30	1.363 ± 0.33	< 0.001
EMax-Shell/A-L9	1.09 ± 0.39	1.72 ± 1.86	0.017
EMin-Shell/A-L9	1.02 ± 0.89	2.10 ± 5.36	0.150
ESD-Shell/A-L9	1.15 ± 0.36	1.52 ± 0.54	< 0.001
E-Shell/A-L9	0.99 ± 0.31	1.34 ± 0.33	< 0.001
E-A/B-L9	3.33 ± 7.41	2.49 ± 1.87	0.497
E-Shell/B-L9	2.29 ± 1.31	2.94 ± 1.63	0.037

### The diagnostic value of single elasticity index with different frequency probes in distinguishing breast malignant tumors

We first performed single-factor ROC curve analysis on each quantitative and semi-quantitative index of different frequency probes. The results showed that EMean-shell-L14 had the highest diagnostic value in the indexes of L14 group (AUC: 0.757, 95% confidence interval: 0.653–0.861), and EMax-shell-L9 had the highest diagnostic value in the indexes of L9 group (AUC: 0.842, 95% confidence interval: 0.758–0.925). Although EMax-shell-L9 showed better performance than EMean-shell-L14, the difference was not significant (P = 0.07, z = 1.812, Figure S3).

### Constructing prediction models for identifying malignant breast tumors based on ultrasound elasticity indexes of L14 and L9 groups

From the elastic ultrasound indexes of L14 and L9 groups, we screened the breast cancer related indexes with non-zero coefficients using LASSO-logistic regression model. The multivariate logistic regression model was established, and the elastic ultrasound indexes were used as independent predictor for breast cancer.

The pathological diagnosis was used as the reference for multivariate logistic regression analysis. The prediction model based on the elastic ultrasound indexes of L14 group was constructed for distinguishing benign and malignant breast masses: logit(P) = -4.43604 + 0.02471×EMax-Shell-14 -1.79902×ESD-Shell/A-L14 + 4.48985×EShell/A-L14 -0.39023×EShell/B-L14 (Table 2). The AUC of the L14 prediction model was 0.810, with the 95CI% of (0.722, 0.898); and the specificity was 72.22%, sensitivity was 78.95%, and accuracy was 75.00%.

**Table 2:** The elastic ultrasound quantitative and semi-quantitative indexes in L14 group chosen for predicting benign and malignant breast masses

	Estimate	Std error	Diagnostic ratio	95%CI lower bound	95%CI upper bound	*P*-value
(Intercept)	-4.4360	1.6621	0.0118	0.0005	0.3078	0.0076
EMax-Shell-14	0.0247	0.0072	1.0250	1.0107	1.0395	0.0006
ESD-Shell/A-14	-1.7990	0.8796	0.1655	0.0295	0.9277	0.0408
EShell/A-L14	4.4898	1.6401	89.1079	3.5798	2218.0653	0.0062
EShell/BL14	-0.3902	0.2503	0.6769	0.4144	1.1055	0.1190

The prediction model based on the elastic ultrasound indexes in L9 group was constructed for distinguishing benign and malignant breast masses: logit(P) = -8.77662 + 0.85510×SRf-B/A-L9 + 0.02142×EMax-A-L9-0.28446×EMin-A-L9 + 1.62844×EMax-Shell/A-L9 + 2.03559×E-Shell/A-L9 (Table 3). The AUC of L9 prediction model was 0.904, with the 95CI% of (0.858, 0.972); and the specificity was 90.74%, sensitivity was 84.21%, and accuracy was 84.04%.

**Table 3:** The elastic ultrasound quantitative and semi-quantitative indexes in L9 group chosen for predicting benign and malignant breast masses

	Estimate	Std error	Diagnostic ratio	95% Confidence interval	P-value
(Intercept)	-8.7766	1.9978	0.0002	0.0000-0.0077	0.0000
SRf-B/A-L9	0.8551	0.3144	2.3516	1.2699–4.3548	0.0065
EMax-A-L9	0.0214	0.0072	1.0217	1.0074–1.0361	0.0028
EMin-A-L9	-0.2845	0.1449	0.7524	0.5664–0.9996	0.0497
EMax-Shell/A-L9	1.6284	1.0219	5.0959	0.6877–37.7610	0.1110

**Fig. 1:**
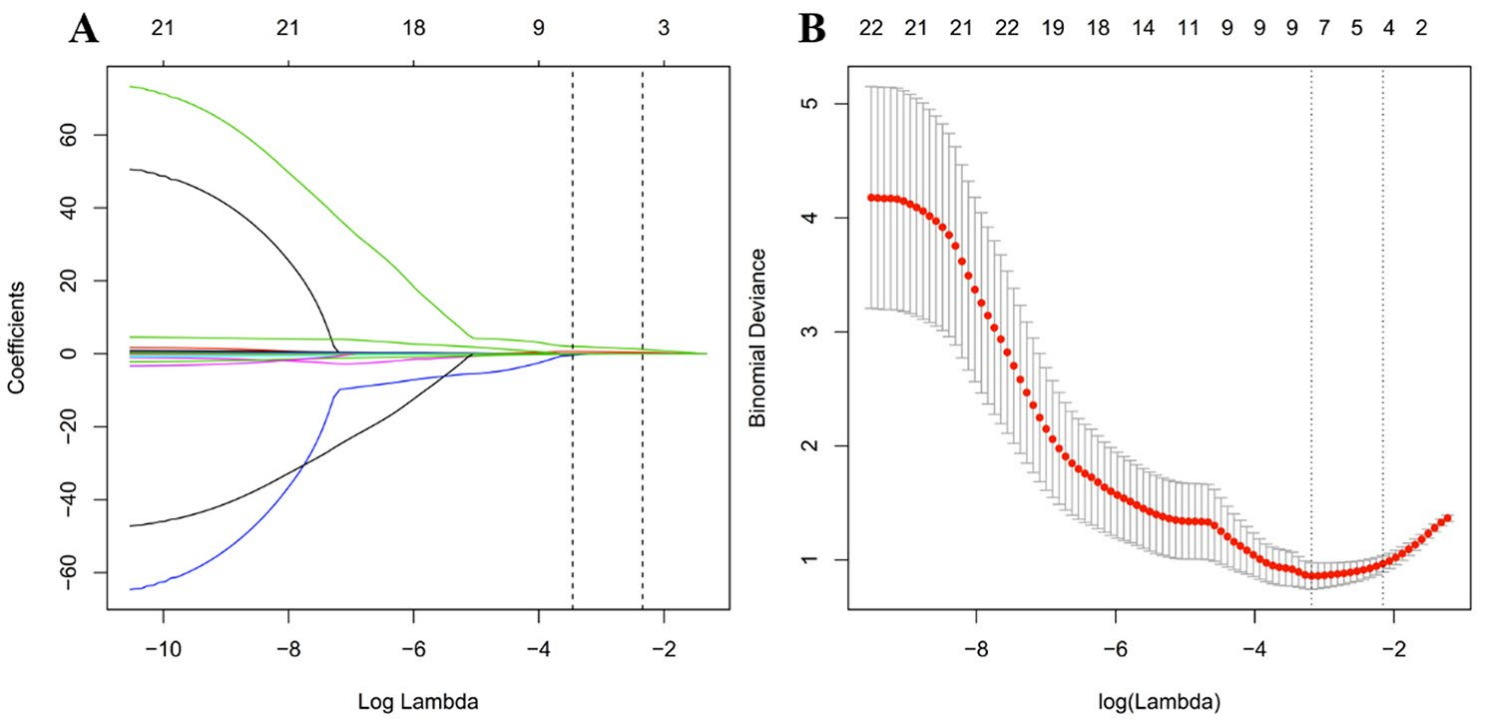
The ultrasonic elasticity index selected using LASSO logistic regression and the accuracy of the prediction model. A, LASSO coefficient profiles of the 18 elastic ultrasound indexes. The dotted vertical line was plotted at the value selected using 5-fold cross-validation in B. The nine resulting features with nonzero coefficients are indicated in the plot. B, Selection of the tuning parameter (λ) in the LASSO model via 10-fold cross-validation based on minimum criteria. Binomial deviances from the LASSO regression cross-validation procedure were plotted as a function of log(λ). The y-axis indicates binomial deviances. The lower x-axis indicates the log(λ). Numbers along the upper x-axis represent the average number of predictors. Red dots indicate average deviance values for each model with a given λ, and vertical bars through the red dots show the upper and lower values of the deviances. The vertical black lines define the optimal values of λ, where the model provides its best fit to the data. The optimal λ value 0.040 with log(λ) =-3.22 was selected

Based on the 92 lesions in the cohort, the 18 features were reduced to 5 potential predictors, which also had non-zero coefficients in LASSO-logistic regression (Fig. 1).

### Comparing the diagnostic efficacies of single index and the two prediction models

**Fig. 2:**
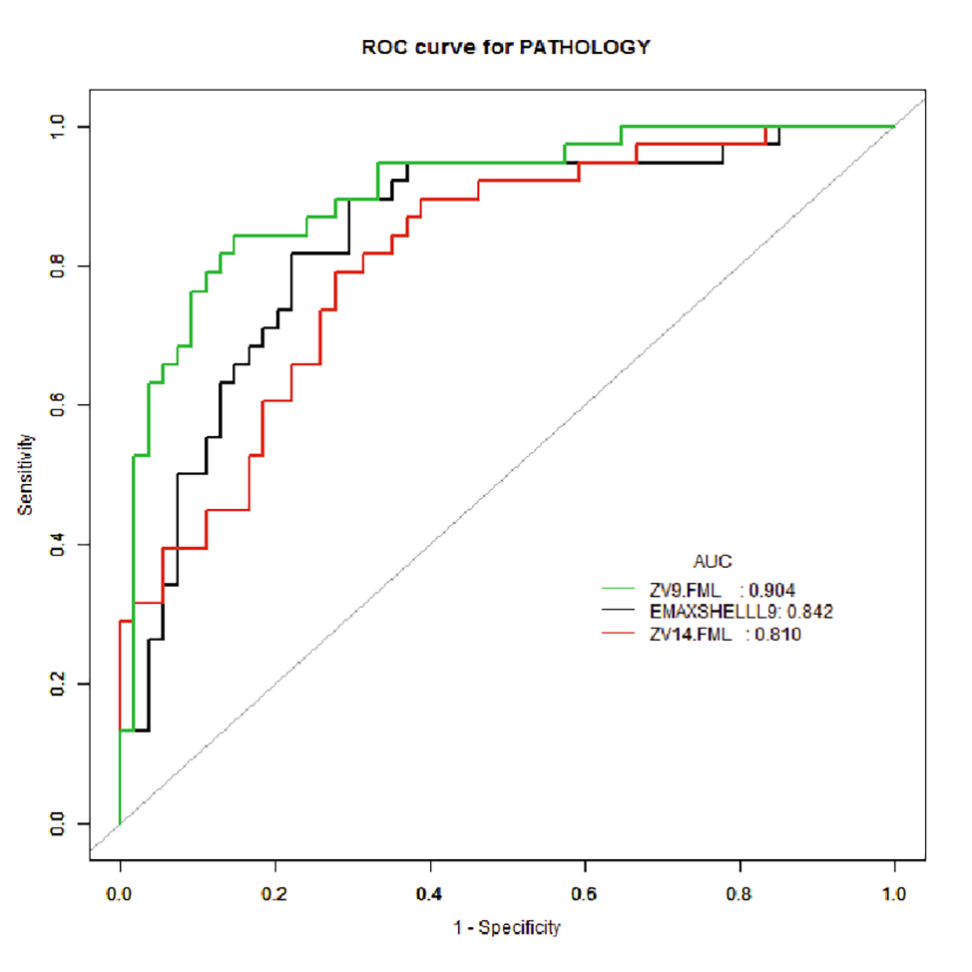
The ROC curves of L9 prediction model, L14 prediction model, and the single index EMax-shell-L9.

**Fig. 3:**
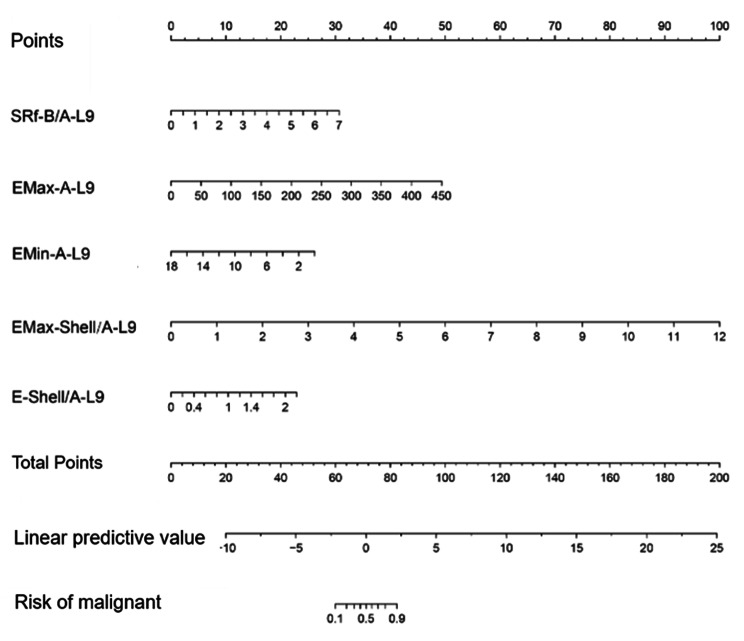
The nomogram of L9 prediction model

ROC curve analysis was used to compare the diagnostic power of the two prediction models. The results showed that the diagnostic value of L9 model was significantly better than that of the L14 model (AUC: 0.905 vs. 0.810, P = 0.0343, z = 2.116), and it was also significantly better than the single index EMax-shell-L9 (AUC: 0.904vs0.842, P = 0.0398, z = 2.056) (Table 4; Fig. 2). The nomogram of L9 prediction model is shown in Fig. 3.

**Table 4:** The diagnostic efficacies of single index and two prediction models

INDEX	AUC	95%CI lower bound	95% CI upper bound	Best cutoff value	Specificity	Sensitivity
EMax-shell-L9	0.842	0.758	0.925	103.055	70.37%	89.47%
L14group prediction model	0.810	0.722	0.898	-0.597	72.22%	78.95%
L9 group prediction model	0.904	0.842	0.967	-0.074	85.19%	84.21%
INDEX	Accuracy	Positive likelihood ratio	Negative likelihood ratio	Diagnostic odds ratio	Positive prediction value	Negative prediction value
EMax-shell-L9	78.26%	3.020	0.150	20.188	0.680	0.905
L14group prediction model	75.00%	2.842	0.292	9.750	0.667	0.830
L9 group prediction model	84.78%	5.684	0.185	30.667	0.800	0.885

## Discussion

The quantitative and semi-quantitative indexes of elastic ultrasound have been widely used in identifying benign and malignant breast masses, and their applications play a significant role in disease diagnosis. The ultrasound frequency commonly used in breast ultrasound examination is 12–14 MHz, but there are few reports on how to select the breast elastic ultrasound frequency. The related reports on the comparison of elastic ultrasound frequencies are mostly in vitro studies comparing convex array low-frequency probes and linear array high-frequency probes [2, 3]. However, since breast is a surface organ, convex array probes are not applicable. Therefore, this study used high-frequency linear array probes with different frequencies to investigate the value of breast elastic ultrasound, and explore which frequency is more suitable for breast elastic ultrasound and more helpful for distinguishing benign and malignant breast masses. Currently, single index of elastic ultrasound is more commonly used for breast mass evaluation, such as the maximum or average value of the breast or surrounding tissues. However, tumors are heterogeneous, and a single index cannot accurately reflect all the tumor characteristics [13]. Therefore, this study used R language (Radiomics) to extract high-dimensional features from the elastic ultrasound data of breast masses, and used nomograms to predict the risk of breast cancer.

In order to ensure the consistency of the study subjects, the two groups used the same facet of the same batch of masses, and each mass was examined by two elastography modes. These two types of elastography did not require manual pressure, and both had their own quality control system, thereby reducing the variation of human operation and increasing repeatability and reliability [14].

Due to the pro-fibrotic reaction inside and around malignant lesions, the malignant lesions were usually harder than benign lesions [15, 16]. Therefore, the modulus of elasticity in malignant lesions was also higher than that in benign lesions, which is consistent with previous study [17]. In the quantitative and semi-quantitative studies of breast elasticity [1, 18–20], the maximum or average value of shear wave velocity or Young’s modulus within the lesion was found to have a higher diagnostic efficiency for benign and malignant breast mass. Our study showed that in sound touch elastography, the diagnostic value of shell was higher than the mass itself, which is consistent with the result from Zhang et al. [1]. The possible reason is that when the surrounding tissue of malignant lesion is too hard, the sound energy attenuation is more severe, and the transverse wave is difficult to propagate in the lesion. Therefore, a false-negative manifestation of low Young’s modulus inside the lesion might occur [17, 21], while the periphery of the lesion presents as a hard ring. Therefore, Shell is more valuable in single factor analysis. In this study, some of the sound touch elastic ultrasound data were statistically significant, and some of the data with different frequencies were also statistically significant. Change et al. found that, in the liver, the measured value from low-frequency C4-1 probe was higher than the high-frequency L9-4 probe, and the difference was significant [22]. This result is consistent with our study, which suggests that appropriate reduction of probe frequency is useful for elastic quantitative analysis. However, Xu et al. believed that frequency had no significant effect on the reference value and cutoff value [23]. But in our study, the index EMax-shell-L9 of the lower frequency L9-3 probe had the highest diagnostic value (AUC: 0.826).

In this study, there was no statistical difference between the two groups in strain elastography (P > 0.05), indicating that the linear array probes with different frequencies had the same value in strain ratio. In strain elastography, the lesion, shell, surrounding normal glands, and fat were all subjected to the same frequency of pressure, and thereby there was no significant difference in the strain ratio between the various tissues.

Among the elastic quantitative and semi-quantitative indexes, the diagnostic efficiency of single index was non-optimal and the specificity was low. In order to further improve the diagnostic efficiency, we used two linear array probes with different frequencies to establish Multi-factor prediction models, respectively, and compared the diagnostic performance of different frequency linear array probes. The results showed that the diagnostic value of elastic ultrasound was related to probe frequency, and the lower frequency L9-3 probe had higher diagnostic value in breast elastic ultrasound (AUC: 0.904 vs. 0.810). This result is opposite to the results from Wang et al. [2], which might be because that Wang et al. performed the module experiment comparing low-frequency convex array probe and high-frequency linear array probe, and the comparable depth of the two was no more than 4 cm; in their comparison, the high-frequency linear array probe (frequency: 14-5 MHz) was better. In this study, the two frequency probes were both high-frequency linear array probes and commonly used in clinical practice; moreover, the mass and gland structures we examined were highly heterogeneous. Under these practical conditions, the lower frequency L9-3 probe showed better performance.

Interestingly, in our study, the tumor itself was selected as the quantitative index rather than the indexes of surrounding tissues, and there was a minimum value of tumor that has never been used in previous reports. This is in contrast to previous reports and the conclusion of our previous study [24, 25]. We think that this may be related to the texture and heterogeneity of the tumor itself. The combination of the maximum and minimum elastic values of the tumor is more suitable for quantifying the texture and heterogeneity of the tumor. Among the quantitative indexes, the hardness of Shell can reflect the invasiveness of malignant tumors to surrounding tissues. In terms of single factor analysis, the hardness of Shell better reflected the true range of malignant tumors than the hardness of tumor itself.

In addition, in this study, no matter the size of the mass and the depth of the location, strain elastography could yield satisfactory images. However, 12 cases were excluded because the L14-5 probe could not obtain stable sound touch images, while the L9-3 probe could yield stable and qualified images for these masses. The long diameter range of these 12 masses was 1.2-4.0 cm, the short diameter range was 0.6–3.1 cm, and the distance between the posterior edge of the mass and the subcutaneous surface was 2.7-3.5 cm. Therefore, the L14-5 probe has limitations in sound touch elastography for deeper masses. This conclusion is consistent with the study from Xue et al. : when sound touch is used to quantitatively evaluate tissue hardness, depth has an influence on the elasticity measurement of the object [26]. Therefore, the L9-3 probe is more suitable for the elasticity inspection of breast masses.

This study also has limitations. The sample size in this study was small, and thus the specific impact of depth on the elasticity value needs to be further explored in following studies. In addition, the diagnostic indexes selected using R language were highly diverse, and it took longer to construct prediction model than a single index. Finally, there were not enough verification groups to verify the effectiveness of combined diagnosis. The next step is to increase the sample size to optimize and verify the prediction model.

## Conclusion

The prediction model based on quantitative and semi-quantitative elastic ultrasound indexes from L9-3 probe exhibited better performance, which could improve the diagnostic accuracy for malignant breast tumors.

## Electronic supplementary material

Below is the link to the electronic supplementary material.

Supplementary file 1. Figure S1 Flowchart showing the patient inclusion process.

Supplementary file 2. Figure S2 Breast invasive ductal carcinoma. a: Two-dimensional ultrasound image b: Colored blood flow image c: Reliability graph of sound touch elastography, with a reliability of 100% (in the box) d: Sound touch elastography image and elasticity value (in the box) e: Sound touch elastography image and elasticity ratio (in the box) f: strain elastography image and strain ratio (in the box).

Supplementary file 3. Figure S3 A: The EMean-shell-L14 had the highest AUC 0.757 in L14 group; B: The EMax-shell-L9 had the highest AUC 0.842 in L9 group.

## Data Availability

The datasets generated and/or analysed during the current study are not publicly available due this study is the summary of the first stage research, and there are follow-up research plans, but are available from the corresponding author on reasonable request.
