# Supplementary figures and images for: The values of elastic quantitative and semi-quantitative indexes measured from different frequencies in the establishment of prediction models for breast tumor diagnosis
Source: BMC Med Imaging. 2022 Nov 15;22:196. doi: 10.1186/s12880-022-00915-1 (PMC9667602; doi:10.1186/s12880-022-00915-1)

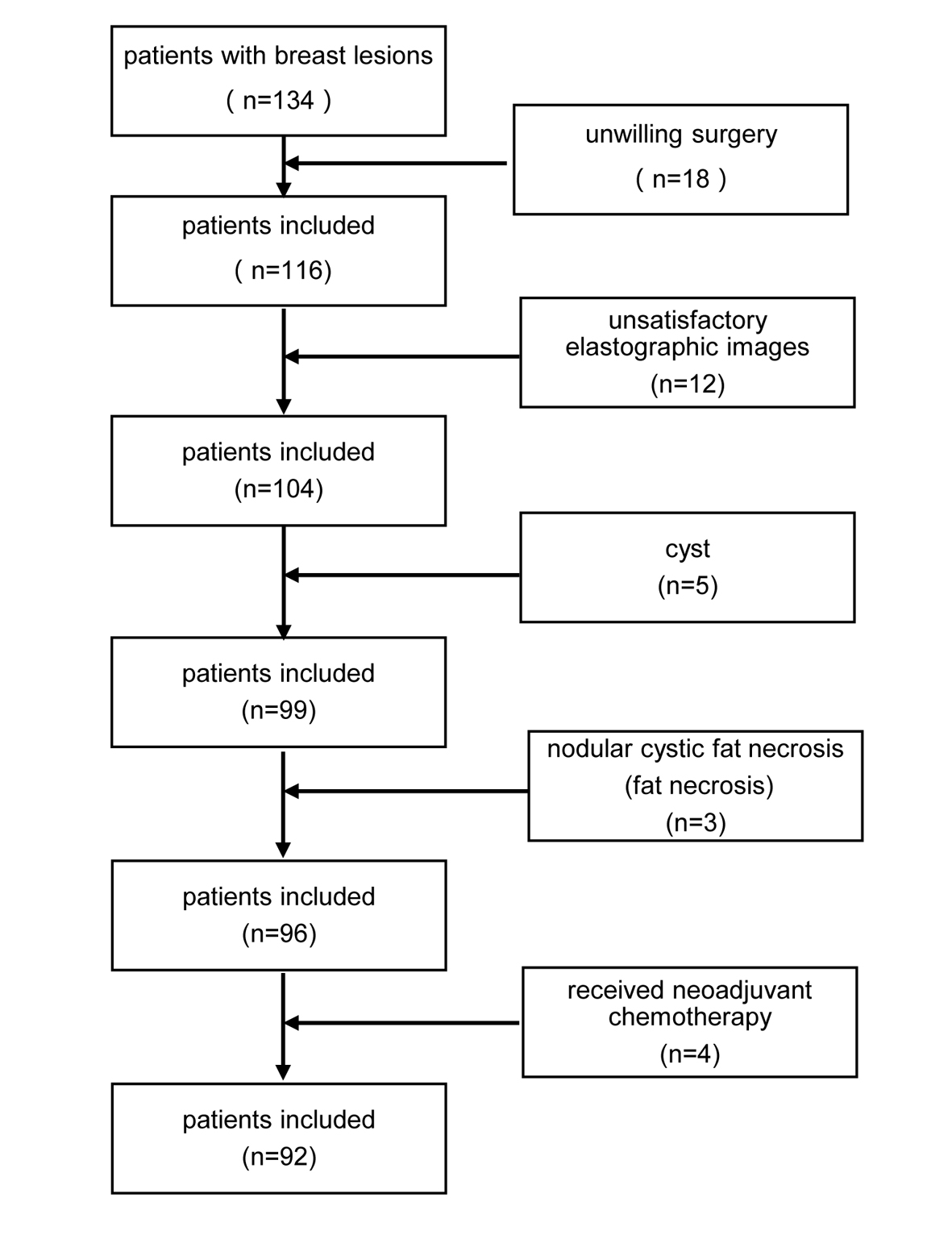

Supplement: Supplementary file 1 — Figure S1 Flowchart showing the patient inclusion process. [file 12880_2022_915_MOESM1_ESM.jpg]

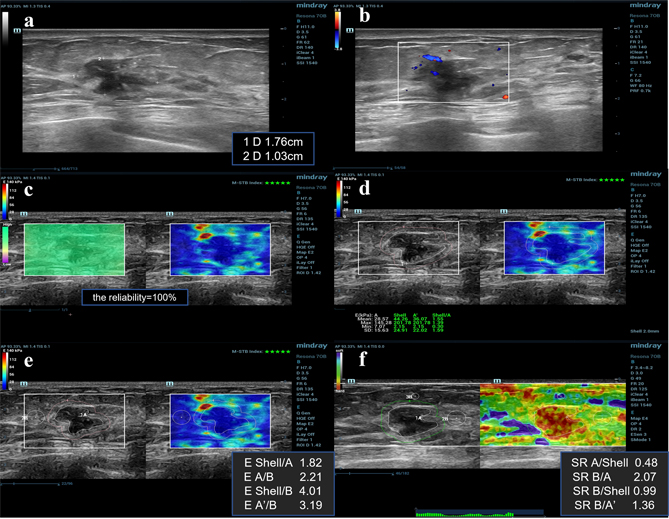

Supplement: Supplementary file 2 — Figure S2 Breast invasive ductal carcinoma. a: Two-dimensional ultrasound image b: Colored blood flow image c: Reliability graph of sound touch elastography, with a reliability of 100% (in the box) d: Sound touch elastography image and elasticity value (in the box) e: Sound touch elastography image and elasticity ratio (in the box) f: strain elastography image and strain ratio (in the box). [file 12880_2022_915_MOESM2_ESM.jpg]

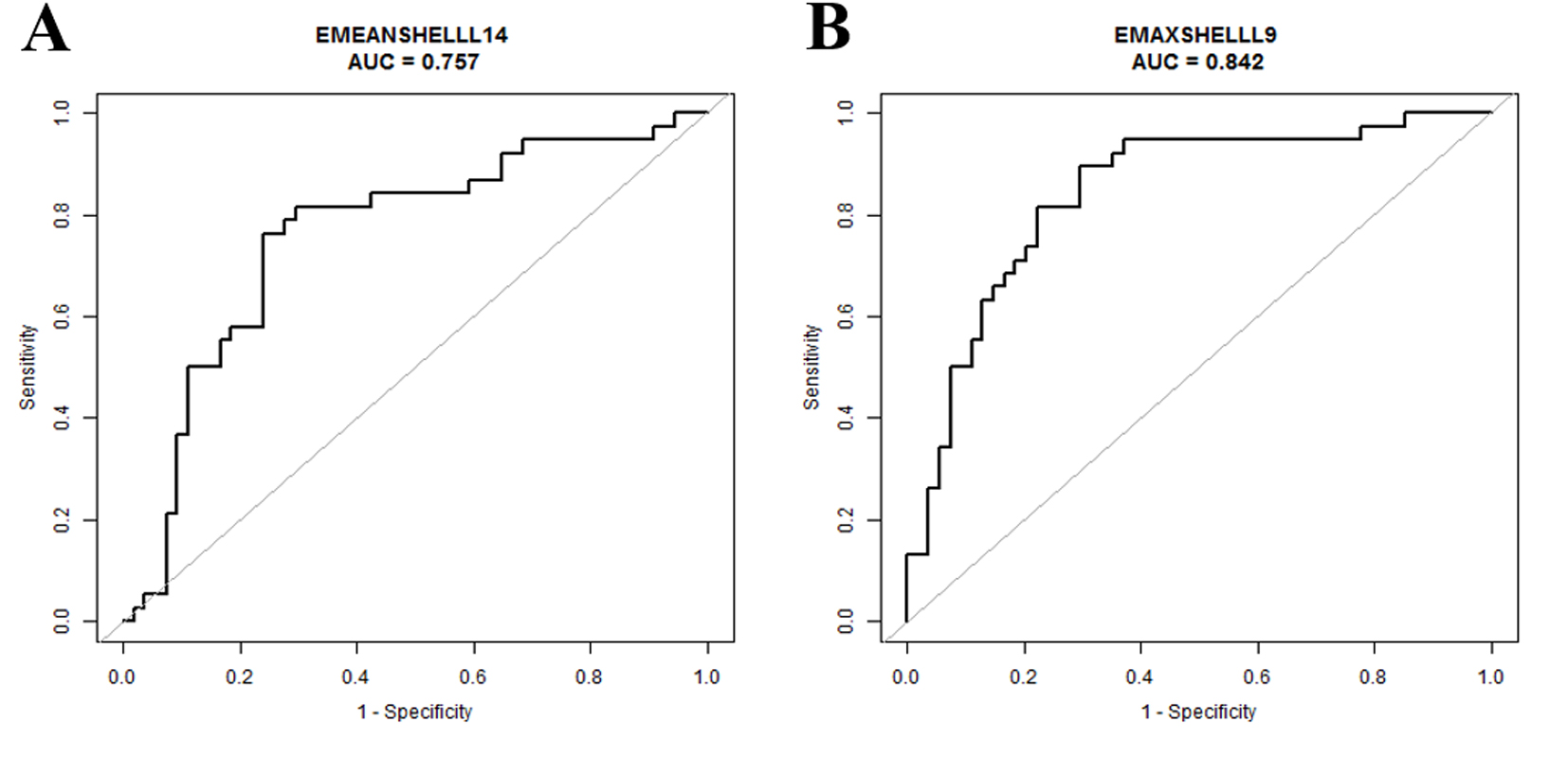

Supplement: Supplementary file 3 — Figure S3 A: The EMean-shell-L14 had the highest AUC 0.757 in L14 group; B: The EMax-shell-L9 had the highest AUC 0.842 in L9 group. [file 12880_2022_915_MOESM3_ESM.jpg]
